# Manifestation of Learner Agency in Primary Education: Goal Setting, Implementation, and Reflection in the Context of Competency-Based Learning

**DOI:** 10.3390/bs15081116

**Published:** 2025-08-18

**Authors:** Jovita Ponomariovienė, Daiva Jakavonytė-Staškuvienė

**Affiliations:** Education Academy, Vytautas Magnus University, 44248 Kaunas, Lithuania; jovita.ponomarioviene@vdu.lt

**Keywords:** learner agency, primary school student, competency-based education, goals, reflection

## Abstract

This article explores the manifestation of learner agency among primary school students within the context of competency-based education. Grounded in social cognitive theory and self-determination principles, the study examines how fourth-grade students set, implement, and reflect on personal learning goals through action research conducted in two Lithuanian schools. A total of 108 students participated by documenting their goals and reflections in journals, while teachers provided insights through interviews. The results reveal that students are capable of demonstrating agency through meaningful goal setting and reflective learning practices, especially when supported by a safe environment, constructive feedback, and opportunities for choice. However, the findings also highlight challenges—such as vague or overly ambitious goals, incomplete reflections, and concerning trends like excessive gaming or emotionally charged goals. The study underscores the critical role of teacher support in scaffolding goal-setting skills and fostering student motivation, while also emphasizing the need for early interventions in emotional literacy and digital well-being. It concludes that learner agency is a developable capacity requiring intentional pedagogical strategies and a reflective school culture.

## 1. Introduction

Contemporary transformations in education, based on the principles of competency-based learning, open up new opportunities not only for content renewal but also fundamentally change the role of the student in the learning process. Competency-based education is grounded in the belief that the student is not a passive recipient of knowledge but an active and responsible participant in their own learning. This paradigm shift calls for a rethinking of what the learning environment should be like—one in which every student can not only acquire knowledge and skills but also develop a conscious attitude toward learning, with the ability to plan, evaluate, and adapt it (OECD, 2019; European Commission, 2018).

In recent years, the concept of agency has been expanded and deepened, increasingly linked with self-regulated learning, autonomous learning, and learning initiative (Jääskelä et al., 2017; Code, 2020; Schoots-Snijder et al., 2025). Learner agency refers to the student’s willingness and ability to act and influence their learning process within a particular sociocultural context. It is expressed when a student is able to set personal goals, choose methods of action, assess their progress, and adjust behavior in response to challenges. Research shows that agentic students tend to demonstrate higher engagement, motivation, and self-confidence, which in turn positively influences their academic achievement and emotional well-being (Code, 2020; B. Zimmerman, 2000). Self-regulated learning theory (Boekaerts, 1996; Pintrich, 2000; B. Zimmerman, 2000; B. J. Zimmerman & Kitsantas, 2002) is understood as an active and conscious process in which students themselves shape their thinking, emotions, and actions in pursuit of personal learning goals. This process is influenced by various environmental factors. Students apply different strategies, such as cognitive or motivational ones, which they select, adapt, and adjust according to their goals and individual characteristics and dispositions. This process takes place within a specific context shaped by broader social and cultural influences—such as family educational practices or school climate—as well as immediate elements, such as task instructions or available resources (Ben-Eliyahu & Bernacki, 2015; Brenner, 2022; Tinajero et al., 2024).

Self-regulation in learning is not merely an innate mental ability or an academic skill—it is an active, intentional, and goal-directed process through which learners transform their knowledge and abilities into effective learning strategies. Self-regulated learners formulate their own goals and consciously monitor their thinking, emotions, and actions in order to achieve those goals (adapted from B. Zimmerman, 2000; B. J. Zimmerman & Kitsantas, 2002; Brenner, 2022; Tinajero et al., 2024).

Recent studies suggest that learner agency may be enacted through self-regulated learning behaviors, such as goal setting, planning, monitoring, and reflection, particularly when students are given the opportunity to make meaningful choices in their learning process (Jääskelä et al., 2017; Code, 2020). Therefore, in this study, self-regulated learning is not treated as separate from agency, but as one of its key expressions within the competency-based education framework.

Based on social cognitive theory, agency is the ability to initiate purposeful action, make autonomous decisions, and regulate one’s behavior in pursuit of meaningful goals (Bandura, 2001). Agency encompasses intention, motivation, self-efficacy, and self-regulation—key components that operate within social and cultural learning contexts (Bandura, 1986; Fletcher, 2016). Within this system of reciprocal interactions, the student acts as an active agent capable of shaping their learning trajectory even under external constraints because it is based on context (Bunn & Lumb, 2019; Hartung, 2011). Children’s responses provide insights into their experiences at school and the learning process, as well as how they perceive learning. The diversity of children’s responses reflects their social and cultural experiences, which shape their beliefs about the learning process, their beliefs about what is important for them to learn, and what might be the best context for learning (Ruscoe et al., 2018).

Agency is not innate—it develops gradually—and fostering it requires specific pedagogical preconditions: a safe and supportive learning environment, authentic student engagement, opportunities to make choices, purposeful feedback, and consistent teacher guidance (Hattie & Timperley, 2007; Anderson et al., 2019; Watling et al., 2021; Schoots-Snijder et al., 2025). In competency-based education, it is essential that students have real opportunities to make decisions about their learning—to set goals, choose how to complete tasks, and evaluate their progress. This educational model not only meets the demands of modern education but also strengthens students’ capacity for lifelong learning and adaptation to constantly changing circumstances (Biesta et al., 2008; OECD, 2022).

Competency-based learning (CBL), as implemented in this study, serves not only as an educational context but also as a foundational framework for the development of learner agency. By emphasizing mastery over time-based progression, CBL encourages students to take initiative, exercise autonomy, and reflect on their learning choices. The alignment between CBL and agency lies in the shared emphasis on student ownership, personalized pathways, and metacognitive engagement. Within such a model, goal setting, feedback processing, and reflection are not supplementary practices but central mechanisms through which agency can emerge and be nurtured (Ryan & Deci, 2016; Code, 2020; European Commission, 2018).

Fostering learner agency is especially significant in primary education, where core learning attitudes and foundational skills are being formed. Although agency is often associated with older students or higher education, a growing body of research shows that even primary school-aged children, when provided with structure, support, and clear goals, are capable of demonstrating signs of agentic learning—making responsible decisions, consciously pursuing goals, reflecting on their progress, and proposing changes (Kiemer et al., 2015; Charteris & Thomas, 2016). However, for such abilities to develop consistently and sustainably, not only an appropriate pedagogical environment is needed, but also teacher professionalism—the ability to respond to individual learners’ needs, personalize instruction, reflect on practice, and act as co-creators in the learning process (Priestley et al., 2015).

Taking these prerequisites into account, this article seeks to analyze the conditions that promote the development of learner agency in the context of competency-based education, based on an action research study in which primary school students independently set their learning goals, planned and monitored their implementation, and reflected on their progress.

### 1.1. Research Aim

To analyze what types of goals fourth-grade students independently set, implement, and reflect on under action research conditions within a competency-based learning context.

### 1.2. Research Objectives

To theoretically substantiate the concept of learner agency and its links with competency-based primary education.

To conduct a qualitative content analysis and describe students’ experiences in setting personal goals, implementing them, and engaging in reflection.

## 2. Literature Review

### 2.1. Conditions That Support the Development of Learner Agency

Fostering learner agency in the context of competency-based education requires intentionally created pedagogical and social conditions. Three key conditions are identified as having a significant impact on students’ ability to act independently, make decisions, and participate responsibly in the learning process: a safe and supportive learning environment, purposeful feedback, and the opportunity for students to make choices and engage in reflection (Bandura, 2006; Watling et al., 2021).

#### 2.1.1. A Safe and Supportive Learning Environment

It is essential to ensure a safe and supportive learning environment where students feel valued, are willing to take risks, make mistakes, experiment, and ask questions. Only when psychological safety is present do children begin to take initiative more actively, set personal goals, and consciously engage in the learning process (Bandura, 2006; Watling et al., 2021). Research shows that such an environment helps meet three fundamental psychological needs of students—competence, relatedness, and autonomy—which are crucial for the expression of learner agency (Ryan & Deci, 2016; Zhou et al., 2019).

A positive learning atmosphere is characterized by emotional safety, social support, trusting relationships between teachers and students, and the freedom to make mistakes and learn from them. Such an environment not only increases students’ self-confidence but also boosts their motivation and active engagement in learning activities (Singh & Priya, 2015; Qureshi et al., 2023; Dalimunthe et al., 2024; Schoots-Snijder et al., 2025). Students are given the space to experiment, make choices, take decisions, and reflect on their progress—all of which form the foundation of agency (Bandura, 2006; Code, 2020).

Moreover, research in educational psychology shows that a safe environment enables the individualization of teaching and responds to the diverse cognitive, emotional, and social needs of learners (Roth & Jornet, 2017; Borich & Tombari, 2021). Therefore, a positive learning environment is not merely a physical space; it is a cultural and emotional context in which the student feels seen, heard, and empowered to act.

#### 2.1.2. Purposeful and Targeted Feedback

A culture of purposeful, targeted, and learning-oriented feedback is critically important. A teacher’s ability to provide individualized, clear, timely, and constructive feedback enables students not only to understand their progress but also to learn from experience, adjust their learning strategies, and build self-confidence (Hattie & Timperley, 2007; Carless & Boud, 2018). Research shows that such feedback enhances student engagement and promotes deeper cognitive processes—such as analysis, decision-making, and reflection—while helping to shape effective learning practices (Winstone & Boud, 2020; Ding, 2025).

When feedback is delivered respectfully and with encouragement, it strengthens learner agency by positioning students as active users of feedback, capable of interpreting and applying it independently (Frattarola et al., 2024; Sanchez-Gil-Machín et al., 2025). In addition, both teacher-led and peer feedback contribute to the development of critical thinking, decision-making, and collaboration skills—key attributes of an active and responsible learner (Nicol & McCallum, 2021; McConlogue, 2015).

#### 2.1.3. Student Choice and Reflection

The development of learner agency requires that students are given opportunities to make choices—not only in setting goals or selecting how to complete tasks, but also in engaging with assessment, making independent decisions, and reflecting on their learning experiences (OECD, 2019). Such opportunities enhance self-efficacy, intrinsic motivation, and empower students to consciously manage their learning (Shaheen et al., 2023; Bubnys, 2019). Reflective learning enables students to connect theoretical knowledge with personal experience, evaluate their actions, and create meaningful change (Colomer et al., 2018; Lefebvre et al., 2023).

In this process, the teacher takes on the role of a mentor—not only encouraging reflection but also helping the student structure it, align it with learning goals, and develop a sense of responsibility (Demkanin, 2022; Connac, 2022). The opportunity to make choices, when grounded in trust, collaboration, and emotional safety, becomes a powerful motivator for students to act independently, ask questions, experiment, and grow both academically and socially (Chan & Lee, 2021).

### 2.2. Setting, Pursuing, and Reflecting on Personal Learning Goals as a Foundation for the Expression of Learner Agency

One of the key expressions of learner agency in competency-based education is the setting, pursuit, and reflection on personal learning goals. Agency involves not only the ability to act independently but also the conscious regulation of the learning process through decision-making, taking responsibility, and self-monitoring (Bandura, 2006; Code, 2020). Goal setting becomes the initial step in strategic learning, in which the student identifies their own objectives, while the subsequent stages—planning, implementing actions, monitoring progress, and reflecting—strengthen intrinsic motivation and foster a sense of self-efficacy (Shaheen et al., 2023; B. J. Zimmerman & Kitsantas, 2002).

Research shows that students who formulate their own goals experience greater satisfaction, engage more deeply in the learning process, and feel more in control of their progress (Ryan & Deci, 2016; Escorcia & Fenouillet, 2018). Goal-setting theory (Locke & Latham, 2006, 2019) also confirms that clear, specific, and measurable goals help guide purposeful action, increase student effort, and enhance learning effectiveness. Particularly important is student commitment to goals, which depends on their level of self-efficacy and the personal significance of the goal (Jeong et al., 2021). Alessandri et al. (2020) argue that the potential of goal setting lies in students’ ability to independently set goals that are appropriately challenging and specific. Difficult, specific goals tend to lead to better outcomes than easy or abstract ones (Latham & Seijts, 2016; de Vreugd et al., 2025).

Self-regulation skills—such as planning, action implementation, monitoring, and strategy adjustment—help students consistently pursue their intended outcomes (Inzlicht et al., 2021). Feedback plays a vital role in this process; it not only identifies progress but also helps to revise goals and tasks (Tolli & Schmidt, 2008). When students receive constructive, motivating, and growth-oriented feedback, they are more likely to set challenging and personally meaningful goals, thus increasing their potential for achievement (Budworth et al., 2015). Learning journals and reflective practices, as additional tools, encourage students to think more deeply about their learning journey, strengthen their awareness of abilities and actions, and support the development of more sustainable learning motivation (Matheson et al., 2017).

Thus, goal setting, implementation, and reflection form an integrated cycle of agentic learning, enabling the student to grow as an active, reflective, and responsible co-creator of their own education.

## 3. Materials and Methods

### 3.1. Contexts of Schools

The study was conducted in two Lithuanian schools (see Table 1). Schools were intentionally selected to analyze educational practices in diverse contexts and situations.

### 3.2. Teacher and Student Context

The study involved six primary school teachers (see Table 2) working in two different schools. The research context included teachers who, as of September 2024, were teaching fourth-grade students. Their lessons were observed during May–June 2024, and in September–October 2024, the teachers participated in interviews. This study is described in a published article (Ponomarioviene et al., 2025).

The research also included the fourth-grade students taught by these teachers. A convenience sampling method was applied. The study was conducted between January and February 2025. Information about the teachers is presented in the table below.

In this study, the teacher–researcher played a role in structuring the learning environment, facilitating student goal setting, and supporting reflective practices. This aligns with the principles of action research, where the practitioner’s involvement and reflective adjustments are integral to the inquiry process.

The study involved 108 students, including 53 boys and 55 girls. As shown in Table 3, there were students who agreed to participate in the study but did not engage in filling out the goal-setting journals and returned them uncompleted. These students were not included in the final count of participants. The students who took part in the study had not previously engaged in setting intentional monthly or weekly goals, nor in monitoring and analyzing their progress toward achieving them. During this study, the students attempted for the first time to set a monthly goal and four weekly goals, and to reflect, analyze, and record their observations in a journal.

### 3.3. Research Method

The chosen research method, action research, was selected because it is considered a democratic, just, and emancipatory qualitative research methodology that promotes an understanding and exploration of real-life contexts (Creswell & Creswell, 2023; Koch & Kralik, 2006). In applying action research, individual emotions, perspectives, and work models are revealed through observation and analysis rather than through control or manipulation. Participants actively engage in making informed decisions throughout the educational process. Additionally, action research enables the identification of emerging issues and their solutions (Soucy et al., 2020; Soucy, 2019). Action research is performed, and there are six principles that characterize it (McConnell et al., 2020; Moosa et al., 2020; Rosen et al., 2020; Mertler, 2021), as follows:

- Purposefulness of the action (the action is performed consciously and purposefully);
- Critical reflection and assessment of the action;
- Cooperation (participants in the action project are also researchers; the researcher is an active participant in the action);
- Orientation to change (actions are flexible and open to modification);
- Cyclicality (four main stages of action research, dynamically linked in a continuous cycle: planning, action, observation, reflection);
- Unity of theory and practice (theory informs practice, practice refines theory, the process is oriented towards change).

### 3.4. Data Collection

The qualitative research was conducted in two stages, as follows:

Stage I. The research data were collected through action research, analyzing the goals set by students, their reflections, and observations, and organizing the data into thematic categories that emerged from the analysis.

#### 3.4.1. Setting Monthly Learning Goals, Planning Steps to Achieve Them, and Reflecting at the End of the Month

**Figure 1:**
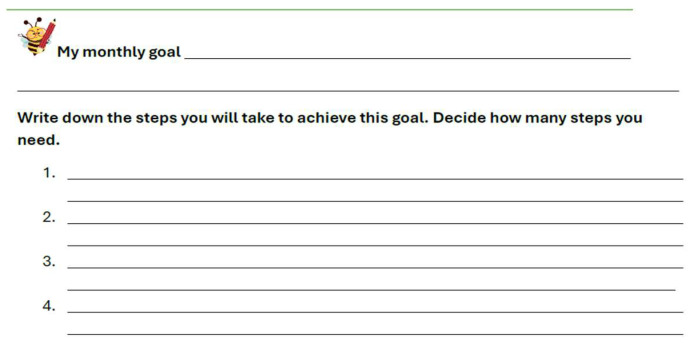
Example of a student form for recording a monthly goal and action steps.

During the study, each student was given a journal titled “Learning to Achieve My Goals”. In this journal, students were asked to write down a monthly goal and outline the steps they would take to achieve it (see Figure 1). As shown in the figure, students were given complete freedom to decide what kind of goal they wanted to pursue and to determine the steps they would take toward achieving it.

**Figure 2:**
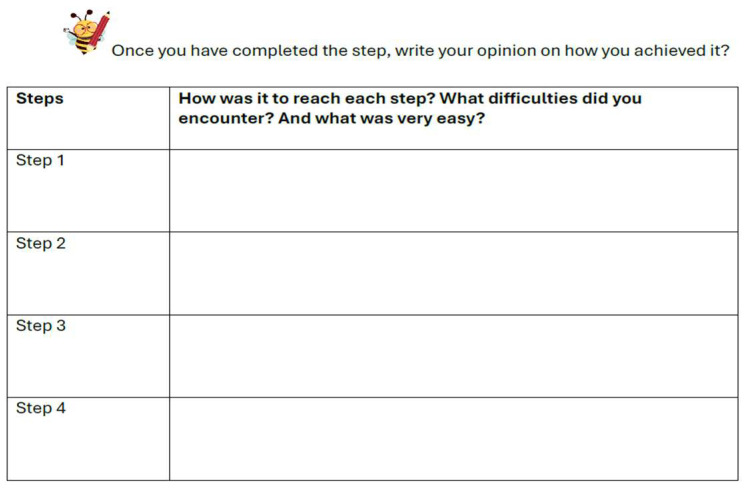
Example of a student form for tracking planned steps toward achieving a goal.

Throughout the month, the students observed themselves and analyzed how successfully they were progressing toward their goal and completing the steps they had planned. They recorded their reflections in a table (see Figure 2).

**Figure 3:**
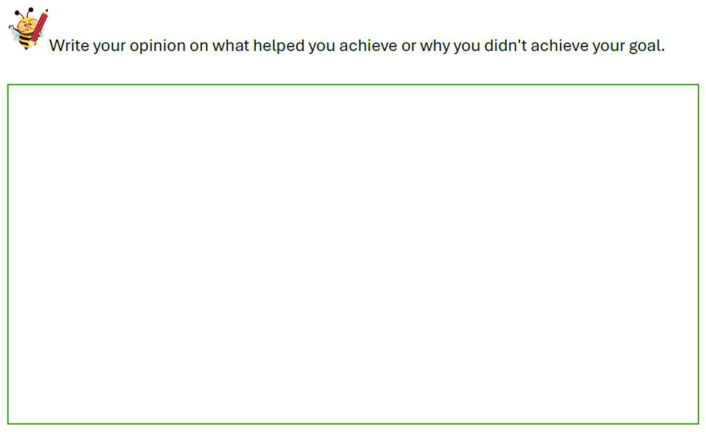
Example of a student reflection form on whether the monthly goal was achieved or not.

After one month, whether they achieved their goal or not, students were asked to write their opinion on why they succeeded or did not succeed in reaching their goal (see Figure 3).

#### 3.4.2. Setting Weekly Learning Goals and Reflecting at the End of the Week

**Figure 4:**
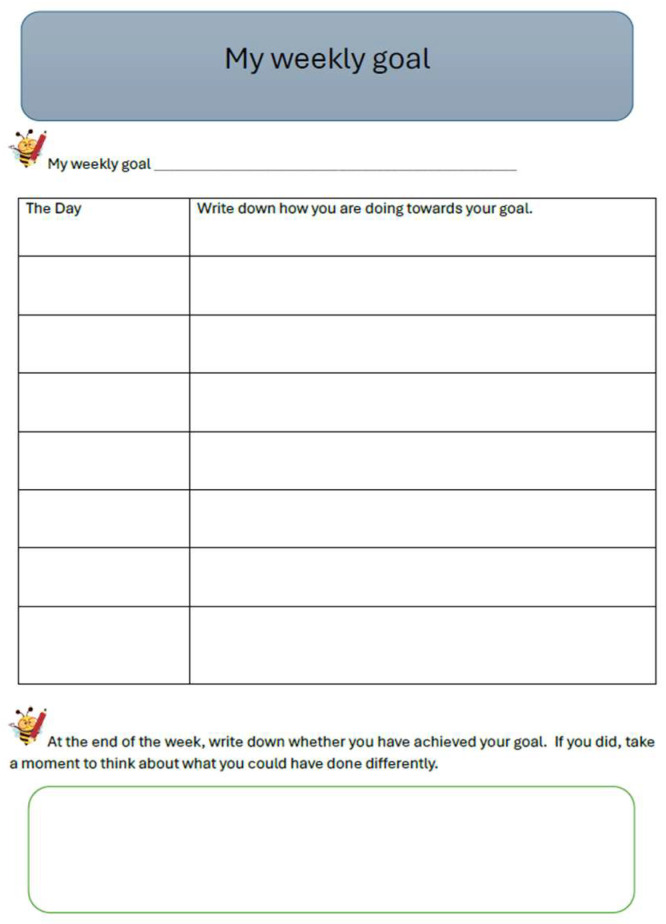
Example of a student form for setting a weekly goal and reflecting on goal achievement.

For four consecutive weeks, students set weekly goals, monitored themselves throughout the week, and recorded their reflections in the journal (see Figure 4). Students were given complete freedom to choose the area in which they wanted to make personal progress during the week. Based on the content and activities planned for the week, as well as their own individual situations and abilities, students identified a weekly goal.

Importantly, during the week, each student reflected and wrote in the journal how they were progressing toward the goal and whether they succeeded in achieving it.

Stage II. To further deepen the data and its analysis, semi-structured interviews (Baškarada, 2014; Yin, 2018) were conducted with all teachers in February 2025. In addition, student focus groups were formed in each class, resulting in six focus groups of five students each.

The analysis of students’ “*Learning to Achieve My Goals*” journals, along with the interviews with teachers and students, created an integrative approach that contributed to a more accurate and comprehensive interpretation and understanding of the research findings.

The interview results were analyzed and systematized using qualitative thematic content analysis, aiming to identify key themes reflected in the responses of both teachers and students. In analyzing students’ monthly and weekly goal setting and their reflections on progress and achievement, recurring patterns, perspectives, and experiences were identified. This not only allowed researchers to name the most common student strategies but also to determine how teachers encouraged students to pursue their goals consistently and what types of support were essential for them (King & Horrocks, 2010; Patton, 2014; Creswell & Poth, 2018; Creswell & Creswell, 2023).

The analysis of student and teacher interviews helped to uncover shared insights as well as key differences and specific aspects of teaching practice that contribute to student development and motivation to set goals, work toward them, monitor their progress, and reflect on their experiences. Moreover, the study highlighted how the process of reflection—combined with teacher support and motivation—impacts students’ reflective abilities, revealing both challenges and effective strategies applied within the school context.

### 3.5. Research Ethics

The study was conducted following the ethical principles of research outlined by Vytautas Magnus University, as approved by the Ethics Committee of the Institute of Educational Research (Resolution No. 2025-17, dated 28 January 2025). Confidentiality requirements were adhered to throughout the study. Students and teachers were informed about the study’s purpose and procedures and their right to withdraw from participation without any consequences. The students’ parents provided individual consent for their children to complete the “*Learning to Achieve My Goals*” journal and for their recorded goals and reflections to be analyzed. Parents also gave consent for their children to participate in the semi-structured interviews. The data are presented with participant responses translated into English.

## 4. Results

In the empirical data analysis section, due to the limited scope of the article, we will present only the results and a summary of the specific monthly and weekly goals that students pursued, how successfully they achieved them, and how they reflected on their progress see Table 4).

The study revealed that students’ monthly goals are diverse and primarily focused on personal development and physical abilities. Sports, artistic activities, and reading remain the main areas dominating students’ aspirations. The largest number of students (*N* = 32) aim to improve in sports by setting clearly measurable physical challenges, such as increasing the number of push-ups or pull-ups, enhancing various skills in different sports, or participating in competitions. This indicates that physical activity is a significant part of students’ daily lives, fostering both personal growth and discipline.

The second most prominent area in which students set personal goals is drawing (*N* = 21). Common objectives include improving drawing techniques, developing the ability to draw specific objects (e.g., animals, 3D buildings, flowers), and maintaining a consistent work schedule by dedicating specific time to drawing weekly or even daily.

The third most popular area is reading books. Sixteen students aim to read more books or further improve their reading skills. Some students set concrete monthly targets for the number of books or pages to read (e.g., 2 or 5 books, 200 pages), while others focus on improving expressive and clear reading. This trend shows that some students have an interest in literature and actively include reading in their monthly activity plans.

Other areas reflect students’ efforts to improve academic achievements. For example, four students aim to improve in mathematics, two in Lithuanian language, and one in a foreign language—Japanese. Five students focus on financial literacy, setting savings or investment goals, which can be considered an important part of personal financial education.

Beyond traditional academic or physical activities, students are also interested in leisure pursuits. Four students aim to improve various hobbies such as solving the Rubik’s Cube, building domino houses, or learning yo-yo tricks. Aspects of healthy living also appear in students’ goals. Two students strive to improve their eating habits by giving up unhealthy food. Additionally, two students seek to develop musical skills—one aims to learn to play the piano, and the other to improve vocal abilities. Meanwhile, two students set goals to advance in dance. Two students focus on computer games and aim to reach higher game levels.

These results show that students’ goals reflect not only academic ambitions but also priorities related to personal development, self-expression, and leisure activities. Their diversity emphasizes the importance of individual interests in the educational process. The identified goals clearly define expectations and areas where efforts should be concentrated (Sedrakyan et al., 2020).

The data in Table 5 reveal changing student priorities in goal setting over the course of four weeks, reflecting trends in motivation, perseverance, and shifting areas of focus. The most consistently chosen goal category was sports, which the largest number of students selected in the first week (*N* = 21), but by the fourth week, the number of students choosing this goal dropped to *N* = 12. This decrease suggests that although physical activity is an attractive initial goal, maintaining engagement may require additional motivation or external reinforcement.

A similar pattern was observed with reading goals, which remained relatively stable during the first two weeks (*N* = 13, *N* = 14) but decreased in the following weeks (*N* = 9, *N* = 8), indicating a possible decline in intrinsic motivation or competing priorities.

Health-related goals, such as healthy eating, decreased sharply from N = 14 in the first week to *N* = 2 in the last week. This suggests that although students recognize the importance of health-oriented behaviors, they may need structural guidance or support systems to maintain long-term commitment.

Comparing monthly and weekly goals, we see that students tend to set short-term goals related to food preparation and cooking (Week 1: *N* = 6, Week 2: *N* = 10, Week 3: *N* = 7, Week 4: *N* = 11), for example, “*Bake an apple pie*” (M7), “*Help my mom tidy the house*” (M4), and “*Cook meals with my mom*” (B10). There are also goals related to handicrafts (Week 1: *N* = 8, Week 2: *N* = 2, Week 3: *N* = 2, Week 4: *N* = 1), such as “*Finish making a plush toy*” (M23), “*Learn to fold origami well*” (B34), and “*String four bead bracelets*” (M45). Additionally, students set goals concerning pet care, TV watching time, emotions, and making new friends.

**Concerning Goals.** The results show that a considerable number of children set goals related to computer games (Week 1: *N* = 12, Week 2: *N* = 8, Week 3: *N* = 8, Week 4: *N* = 5). Examples include the following:

*“Save up to 60 money in the game” (M5), “Build the most beautiful house in the game” (M10), “Get 45 points in the game ‘Brawl Stars’” (B6), “Improve at Fortnite” (B12), “Play Roblox all day” (B43), “Try to get mom to increase the internet so I can play games” (B48), “Earn 50 money in the game” (B2),*

*“Play the game ‘Scratch’” (B34), “Get 41 kills with Dynamike in the game” (B41).*

The researchers analyzed the daily reflections of student B12, who set the goal “*Improve at Fortnite.*” The student recorded the time spent playing each day, as follows: Monday—3 h, Tuesday—2 h 40 min, Wednesday—4 h 30 min, Thursday—3 h 40 min, Friday—5 h 50 min, Saturday—6 h 50 min, and Sunday—11 h 54 min. These data show that the number of hours spent playing computer games is especially high during the weekend. This raises concerns about students’ conscious time management and the impact of computer games on their daily lives.

Only one student attempted to limit computer time, setting the goal: “*I will try to play computer games not more than 1 or 2 h instead of 3 h*” (B33). In the daily reflection, the student noted the following play times: Monday—1 h, Tuesday—1 h 30 min, Wednesday—1 h, Thursday—2 h, Friday—1 h 30 min, and Saturday and Sunday—2 h each.

Considering the scale of digitalization in modern society, computer game addiction is becoming an increasingly relevant issue for children’s psychological and cognitive development. While computer games may be valued as a means of socialization, entertainment, or even skill development, increasing research reveals their potential negative impact on children’s attention, memory, and learning abilities. Excessive gaming can lead to attention disorders, weaker working memory, lower learning efficiency, and in some cases, addiction symptoms such as impaired behavioral control, prioritizing gaming over daily activities, and an inability to reduce gaming time despite negative consequences (Nogueira et al., 2019; Farchakh et al., 2020; Han et al., 2022; Imataka et al., 2022; Anjum et al., 2024).

Scientific research also shows that the development of a gaming addiction is influenced by genetic factors, individual psychological characteristics, and environmental context (Choi et al., 2019). It is important to note that a particularly vulnerable group includes children whose self-regulation mechanisms are still developing and whose motivational systems are highly sensitive to external stimuli such as the rapid rewards and stimulating content typical of many computer games.

Growing concern among parents and educators is based not only on psychological or behavioral issues but also on noticeable changes in academic performance. Many sources agree that computer game addiction can impair children’s ability to concentrate, maintain a consistent learning routine, and persistently pursue learning goals (Evren et al., 2019; Stavropoulos et al., 2019).

From the table data, we observe that some students set goals related to death or deceased persons. One student (M32) set the goal “*I want to die*” in week 2, “*Get the heads of the deceased as gifts*” in week 3, and “*Visit grandmother in heaven*” in week 4. Another student (B24) set the goal “*A tough week ahead, I wish I would die,*” and student M53 set the goal “*Kill everyone who bothers me.*” However, upon reviewing these students’ daily reflections, it was noted that none completed or wrote whether they achieved their goals. This suggests that some students may be experiencing emotional difficulties or seeking to draw attention to their internal struggles. A particularly concerning finding was that some students formulated goals related to excessive video gaming or even mentioned themes involving death and aggression. While these instances were not predominant, they raise important questions about the emotional states of learners and the broader conditions in which agency is exercised. In the context of agency theory, emotional experience plays a significant mediating role: negative emotions such as anxiety, frustration, or hopelessness can undermine a learner’s sense of control and efficacy, making it harder to set meaningful goals or persist toward them (Bandura, 2006). Conversely, persistent failure to experience agency—such as not being heard, supported, or successful—can itself intensify distress and lead to disengagement or maladaptive coping behaviors (B. Zimmerman, 2000; Jääskelä et al., 2017).

These emotional dimensions of goal setting and reflection suggest that agency should not be viewed in purely cognitive or behavioral terms. Rather, it is an embodied, emotional experience that develops in response to both internal states and external conditions. Students who set goals focused on avoiding distressing experiences (e.g., reducing screen time due to tiredness or improving sleep) may be demonstrating an emerging awareness of the costs of their behavior, even if the framing is incomplete. On the other hand, references to aggression or death, however isolated, may reflect deeper emotional struggles that interfere with the capacity to engage constructively in learning. These findings underscore the importance of integrating emotional literacy and psychological safety into agency-building pedagogies. Emotional regulation, social support, and a trusting school environment are essential to ensure that all students—not only those who already feel confident—can participate in goal-setting, reflection, and agentic learning processes (Code, 2020; Jääskelä et al., 2017; Schoots-Snijder et al., 2025)

This is a serious signal that the school community should take additional steps—strengthening emotional health support mechanisms, dedicating more attention to individual conversations with students, and providing psychological assistance.

Analyzing the students’ reflections and goal achievement data reveals several important trends. The majority of students (*N* = 67) reflected on their goal pursuit process, indicating reasonably good skills in self-reflection and self-monitoring. This may be related to the school culture, which encourages self-awareness and goal-directed analysis.

Forty-two students achieved their goals, suggesting that most students likely set realistic and attainable goals and had sufficient motivation and resources to accomplish them (Locke & Latham, 2006, 2019). Examples of student comments include the following: “*I set a fairly easy goal and my drawing skills are good*” (M2), “*I managed to achieve my goal. What helped was that I honestly filled in the table every day, and the book was on the table reminding me to draw flowers*” (M6), “*I succeeded because I worked diligently and didn’t skip a single day*” (B2), “*I succeeded because I followed the steps*” (B3), “*I achieved my goal because I tried very hard*” (B15), “*I succeeded because I played less on my phone and computer*” (B19), “*Motivation helped me*” (B44), and “*What helped me achieve the goal is that I am responsible*” (M53).

Meanwhile, 25 students did not succeed in achieving their goals. This may indicate that some goals were overly ambitious or lacked a clear action plan for attainment. Students cited various reasons for not reaching their goals, as follows: “*There was too little time to achieve the goal*” (M17), “*I only completed the first step and wrote down two steps, but I couldn’t focus and failed to complete the second*” (M30), “*I was bored, so I didn’t do it*” (M31), “*Failed because I forgot many times*” (M33), “*The children distracted me*” (M41), “*I didn’t achieve the goal because I didn’t have time to play tennis*” (B34), and “*I failed because I couldn’t find time*” (M44).

This number raises the question of how teachers and mentors might better assist students in planning their actions and monitoring their progress.

Particular attention is required for the 35 students who did not complete the information about goal achievement. This suggests possible insufficient engagement in the goal pursuit process.

Additionally, Table 6 shows how children formulate goals. Some goals are very general, such as “*Become a professional swimmer*” (M16), “*Be better at basketball*” (B41), “*Learn how to study better*” (M27), “*Study diligently*” (M41), and “*Know Lithuanian well*” (M33). Such goals, lacking clear criteria, can be difficult to achieve.

These data (see Table 6) show that although most students actively pursue their goals, their reflection and goal achievement analysis skills can still be strengthened for better future outcomes. Reflection helps students achieve learning outcomes (Schnepp & Rogers, 2022).

## 5. Discussion and Conclusions

The findings of this study reveal that the ability of primary school students to set, pursue, and reflect on learning goals is a dynamic and evolving process, closely tied to the development and expression of learner agency within a competency-based education context. Consistent with theoretical perspectives (Bandura, 2006; Code, 2020), learner agency is not simply the capacity to act independently; it also encompasses intentionality, motivation, self-efficacy, and the conscious regulation of behavior. This study confirms that even young learners, when supported by a structured learning environment and guidance, are capable of demonstrating key elements of agentic behavior—setting personally meaningful goals, monitoring their actions, and reflecting on their learning.

At the same time, the findings highlight significant variability in how students formulated their goals and engaged in reflection. Many goals were vague or overly abstract (e.g., “study better,” “become a professional athlete”), and reflections were often incomplete or superficial. These patterns suggest that learner agency is still in formation at this age and requires explicit teaching and scaffolding. The data echo Ryan and Deci’s (2016) view that intrinsic motivation flourishes when learners experience competence, relatedness, and autonomy. Students who succeeded in achieving their goals often cited deliberate effort, careful planning, and emotional support, indicating that agency is enhanced when these psychological needs are met. At the primary level, agency is still emerging rather than fully formed. Even when learners struggled to express their thoughts in structured ways, they frequently demonstrated early signs of agency—such as forming intentions, noticing changes in behavior, or articulating emotional responses to success or difficulty. These partial reflections reflect not a lack of agency, but agency in formation. Therefore, they should be recognized as essential components of the learning process, during which students begin to take ownership of their goals and learning experiences.

However, some students did not engage in the reflection process at all. This may reflect low self-efficacy (B. Zimmerman, 2000; Bandura, 2006) or the absence of a reflective culture within the learning environment. As Lefebvre et al. (2023) point out, reflection skills develop progressively when teachers actively guide learners in structuring their thinking and linking experience to goals. Without such support, learners may struggle to see value in reflection or may lack the tools to do so effectively. While this study primarily focused on goal-directed behavior as evidence of learner agency, it is important to acknowledge that non-engagement or resistance may also represent agentic expressions, particularly when learners consciously reject imposed goals or expectations. In the data, some students chose not to reflect or set goals, and while this may reflect low self-efficacy or limited understanding, it may also indicate a form of agency expressed through disengagement.

Feedback emerged as a particularly important element in fostering agency. Both internal feedback—developed through students’ own reflections—and external feedback from teachers played a role in helping students make sense of their learning process. Those who received clear, respectful, and encouraging teacher feedback were more likely to revise their strategies, persist in the face of difficulty, and take ownership of their goals. This supports Hattie and Timperley’s (2007) emphasis on the formative power of feedback, and aligns with Carless and Boud’s (2018) notion that students need support to develop “feedback literacy” through sustained interactions with educators.

The study also revealed several concerning trends related to students’ emotional well-being and the expression of learner agency. Some students set goals involving excessive video gaming, while a few expressed thoughts related to death or aggression. These instances raise serious concerns about children’s emotional health and emphasize the need to strengthen emotional literacy education in schools, ensure access to psychological support, and monitor students’ overall well-being more attentively (Han et al., 2022; Imataka et al., 2022; Anjum et al., 2024).

It is important to distinguish between two separate patterns observed in the data. In some cases, students’ goals referred to the excessive use of computer games, which may indicate diminished attention and self-regulation skills. When most cognitive resources are consumed by gaming, there is little capacity left for setting meaningful goals, planning actions, or engaging in reflection. These tendencies align with research findings showing that excessive screen or game exposure in childhood can impair self-control, attention span, and behavior (Han et al., 2022; Anjum et al., 2024).

In contrast, a few students expressed reflections or goals related to death or aggression. Such statements may indicate deeper emotional or psychological distress and require a different, more individualized pedagogical and psychological response. These patterns are not only signs of low emotional literacy, but may also reflect a lack of support in the school environment. As Lefebvre et al. (2023) point out, emotional reflection and self-reflection skills develop gradually when teachers actively help students structure their thinking and connect experiences to goals. Without such support, students may lack both the motivation and the tools needed to engage in meaningful reflection and agentic behavior.

While both patterns influence how learner agency is expressed, their causes and implications differ. One stems from overstimulation or cognitive overload, the other from internal distress. Therefore, fostering student agency requires taking into account the nature of these challenges and applying differentiated support strategies grounded in both psychological and educational research (Code, 2020; Ryan & Deci, 2016; Bubnys, 2019).

Overall, the results affirm that learner agency is a developable competency that emerges through intentional educational design. Goal setting, implementation, and reflection function as interrelated elements of the agentic learning cycle and must be nurtured through pedagogical approaches that emphasize student engagement, responsibility, and collaboration (B. J. Zimmerman & Kitsantas, 2002; Bubnys, 2019). Yet the variability and fragility of agency observed in this study remind us that agency should not be assumed as a given or treated as a linear outcome. Rather, it is a fluid and context-dependent process shaped by both individual and environmental factors.

In conclusion, this study confirms that learner agency is a developable capacity that can be observed even in early learners through goal setting, reflection, and planning. However, our findings also point to the complexity and fragility of agency, particularly in a primary school context. Agency should not be viewed only through the lens of self-regulation or intentional goal achievement. It is a broader, more fluid construct that can also manifest in less visible or even contradictory ways—such as resistance, avoidance, or emotional withdrawal. These behaviors, often dismissed as disengagement, may in fact be active agentic responses to the constraints of the learning environment or to misalignment between learners’ internal motivations and external expectations (Biesta & Tedder, 2007; Priestley et al., 2015).

Thus, agency must be understood as a relational and dynamic process that emerges over time, shaped by the interplay between individual characteristics, social interactions, and cultural contexts. Rather than aiming to produce a “final self,” education should cultivate spaces where learners can safely experiment with multiple ways of being, acting, and relating to their learning. In such environments, both participation and non-participation may become meaningful expressions of agency. This perspective opens up richer avenues for supporting learners—not just in their achievements, but in their self-understanding and sense of belonging.

## 6. Limitations and Directions for Future Research

This study has several limitations that are important to consider when interpreting the results and planning further research.

The aspects identified and analyzed during the study—related to students setting, pursuing, and reflecting on personal goals within the context of competency-based education—provide valuable insights for both researchers and practitioners aiming to implement educational practices that strengthen learner agency. However, these insights are limited to the specific context of this study.

The study was conducted using a qualitative action research method, so its results cannot be generalized to the entire population. To validate and expand on the findings, it would be advisable to conduct a quantitative study with a larger sample in the future. Such data would allow statistically supported analysis of the relationships between goal setting, progress monitoring, reflection, and learning outcomes, as well as identification of factors that most strongly contribute to the expression of agency. Furthermore, it would be worthwhile to study more deeply the role of teachers in different contexts and how their methods influence student motivation and autonomous learning.

## Figures and Tables

**Table 1:** Contexts of schools in the empirical research.

School	Location	Number of Students in the School	Number of Teachers Who Participated in the Study	Number of Students Who Participated in the Study	School Context
1 school	In a big city	940	4	84	The school serves students in grades 1 through to 8. About 15% of the students have special educational needs. Additionally, approximately 30% of the students come from foreign, non-native speaking, or mixed-language families, where the native language is not the language of instruction at the school. In response to this situation, Lithuanian language instruction is organized by dividing the class into two smaller groups, allowing for more individualized attention and support for students to learn the Lithuanian language.
2 schools	In a small town	191	2	23	The school serves students from the pre-primary group through to grade 12. Class sizes are small, with no more than 15 students per class. The school is located in a small town where the majority of the population are non-native speakers. Although students attend a Lithuanian-language school, for most of them, Lithuanian is not their first language.

**Table 2:** Information about the teachers who participated in the study.

Teachers	Qualification	The Total Work Experience in the School
T1	Qualification of the primary school teacher	27 years
T2	Qualification of the primary school teacher	36 years
T3	Qualification of the primary school teacher	32 years
T4	Qualification of the primary school teacher	22 years
T5	Qualification of the primary school teacher	28 years
T6	Qualification of the primary school teacher	35 years

**Table 3:** Information about the students who participated in the study.

Teachers	Number of Students in the Class	Number of Participating Boys	Number of Participating Girls	Number of Students Who Returned Uncompleted Journals
T1	24	9	14	1
T2	24	11	10	3
T3	20	9	11	0
T4	21	13	7	1
T5	15	5	9	1
T6	14	6	4	4

**Table 4:** Distribution of students’ monthly goals by area.

Categories	Number of Students	Examples of Student Goals (Original Wording Preserved)
Sports	*N* = 32	“Learn to perform the gymnastics move ‘the ring’” (M3) “Do one one-arm push-up” (B6) “Do 15 pull-ups” (B7) “Do morning exercises” (B8), (M30) “Become a professional swimmer” (M16) “Kick the ball from one goal to the other” (B10) “Win karate competitions and get a trophy” (M21) “Earn an orange belt in karate” (B15) “Make 30 layup shots” (B20) “Exercise more” (B13) “Learn to lift my legs high because I do karate and I can’t lift them high” (M29) “Try to achieve more in horseback trotting” (M38) “Create my own martial art ‘Kenji’” (B44) “Learn to skate with ice skates” (M52) “Exercise every week” (B46), (B39), (M14) “Do 50 push-ups” (B43) “Play football better” (B27), (B9), (B16), (B22), (B29) “Juggle the ball more times” (M12) “Learn to play tennis better in a month” (B34) “Practice doing a bridge from standing position every day” (M44) “Learn to do the splits” (M45), (M48) “Do 8 pull-ups” (M47) “Learn to catch the ball” (B40) “Be better at basketball” (B41)
Drawing activities	*N* = 21	“Learn to draw robots” (B3) “Learn to draw animals more beautifully” (M2) “Learn to draw nicely” (M10) “Improve my drawing skills” (M11) “Learn to draw better” (B17), (B25) “Draw animals better” (M22) “Draw 4 pictures” (M23), (M36) “I want to learn to draw beautifully” (M24) “Learn to draw neatly” (M25) “Learn to paint a picture beautifully and carefully” (M28) “Learn to draw 3D buildings” (M43) “Draw 10 pictures in a month” (B33) “Draw 3 pictures every week” (B47) “Learn to draw a cat nicely” (M51) “Learn to draw flowers and a rose” (M6) “Learn to draw a realistic rabbit in a month” (M42) “I will improve my drawing in a month” (B38) “Draw every week” (B45) “Draw a picture every day and improve” (B49)
Reading books	*N* = 16	“Read 2 books in a month” (M7) “Learn to read expressively and clearly” (M1) “Find a good book and read it” (B5) “Read a book” (B18), (B19) “Read 2 books with 200 pages” (M8) “Read more books” (M15) “Read the book *Hans Christian Andersen’s Fairy Tales*” (B32) “Read 5 books (at least 110 pages—at most 170)” (B42) “Read one book in a month” (M39) “Read one book and write a summary in a month” (M20) “Read an entire book in a month” (M26) “Read a book in a month” (B30) “Read 2 books in a month” (M40), (B36) “Read the whole book” (M49)
Finance	*N* = 5	“Fill up my piggy bank within a month” (B4) “Save 30 euros” (B11) “Save 1000 euros” (B28) “I want to learn how to invest money” (B48) “Save money for a new board game” (M32)
Learning how to learn	*N* = 2	“Learn how to study better” (M27) “Study diligently” (M41)
Doing homework	*N* = 1	“Do my homework every time” (M4)
Leisure activities	*N* = 4	“Solve a Rubik’s Cube” (B23) “Solve a Rubik’s Cube in one minute” (B24) “Build a small house out of dominoes in a month” (M46) “Learn 5 yo-yo tricks” (B14)
Learning mathematics	*N* = 4	“Learn to solve word problems in math better” (M31) “Multiply by hundreds to pass the final test” (M34) “Learn math” (B21) “I want to learn to calculate better” (B2)
Healthy eating	*N* = 2	“Not eat unhealthy food” (M9), (M13)
Music	*N* = 2	“Train my voice” (M5) “Learn to play a short piece on the piano” (M35)
Computer games	*N* = 2	“Be a Fortnite genius” (B12) “Reach level 50 in the game” (B1)
Learning Lithuanian	*N* = 2	“Know Lithuanian well” (M33) “Learn all past tenses” (B31)
Dance	*N* = 2	“Learn at least one dance” (M37) “Win a dance championship” (B26)
Learning foreign languages	*N* = 1	“Study Japanese for 5 min every day” (B35)
Emotions	*N* = 1	“Control my emotions when things are difficult” (M18)
Creative works	*N* = 2	“Publish a comic this month” (M17) “Create a three-minute animation” (M50)
Other activities	*N* = 3	“I want to get a dog” (B37) “Learn to bake a pizza” (M53) “Stop biting my nails” (M54)

**Table 5:** Distribution of students’ weekly goals by area.

Categories	Week 1	Week 2	Week 3	Week 4
Sports	*N* = 21	*N* = 18	*N* = 15	*N* = 12
Reading books	*N* = 13	*N* = 14	*N* = 9	*N* = 8
Drawing activities	*N* = 8	*N* = 13	*N* = 3	*N* = 6
House chores and cooking	*N* = 6	*N* = 10	*N* = 7	*N* = 11
Healthy eating	*N* = 14	*N* = 2	*N* = 5	*N* = 2
Computer games	*N* = 12	*N* = 8	*N* = 8	*N* = 5
Learning foreign languages	*N* = 4	*N* = 0	*N* = 2	*N* = 0
Handicrafts	*N* = 8	*N* = 2	*N* = 2	*N* = 1
Sleep routine	*N* = 2	*N* = 2	*N* = 2	*N* = 1
Leisure	*N* = 4	*N* = 3	*N* = 2	*N* = 4
Dance	*N* = 2	*N* = 1	*N* = 0	*N* = 2
Music	*N* = 3	*N* = 2	*N* = 1	*N* = 2
Mathematics	*N* = 1	*N* = 4	*N* = 7	*N* = 7
Emotions and feelings	*N* = 1	*N* = 0	*N* = 0	*N* = 0
Time spent watching TV	*N* = 1	*N* = 0	*N* = 0	*N* = 0
Creativity	*N* = 0	*N* = 3	*N* = 0	*N* = 0
Making new friends	*N* = 2	*N* = 1	*N* = 0	*N* = 0
Pet care	*N* = 1	*N* = 1	*N* = 0	*N* = 2
Doing homework	*N* = 0	*N* = 4	*N* = 1	*N* = 0
Behavior at school	*N* = 0	*N* = 1	*N* = 4	*N* = 2
Goals related to death or deceased people	*N* = 0	*N* = 2	*N* = 2	*N* = 1
Classroom decoration	*N* = 0	*N* = 0	*N* = 4	*N* = 1

**Table 6:** Number of students who achieved or did not achieve their monthly goals.

Number of Students Who Reflected on How They Achieved Their Goal	Number of Students Who Succeeded in Achieving the Goal	Number of Students Who Did Not Succeed in Achieving the Goal	Number of Students Who Did Not Complete Information About Goal Achievement
*N* = 67	*N* = 42	*N* = 25	*N* = 35

## Data Availability

The data that support the findings of this study are available from the corresponding author [Daiva Jakavonytė-Staškuvienė], upon reasonable request.
